# 3D Printed Dry EEG Electrodes

**DOI:** 10.3390/s16101635

**Published:** 2016-10-02

**Authors:** Sammy Krachunov, Alexander J. Casson

**Affiliations:** School of Electrical and Electronic Engineering, The University of Manchester, Manchester M13 9PL, UK; sammy.mahdi@student.manchester.ac.uk

**Keywords:** electroencephalography (EEG), dry electrodes, 3D printing, personalized healthcare

## Abstract

Electroencephalography (EEG) is a procedure that records brain activity in a non-invasive manner. The cost and size of EEG devices has decreased in recent years, facilitating a growing interest in wearable EEG that can be used out-of-the-lab for a wide range of applications, from epilepsy diagnosis, to stroke rehabilitation, to Brain-Computer Interfaces (BCI). A major obstacle for these emerging applications is the wet electrodes, which are used as part of the EEG setup. These electrodes are attached to the human scalp using a conductive gel, which can be uncomfortable to the subject, causes skin irritation, and some gels have poor long-term stability. A solution to this problem is to use dry electrodes, which do not require conductive gel, but tend to have a higher noise floor. This paper presents a novel methodology for the design and manufacture of such dry electrodes. We manufacture the electrodes using low cost desktop 3D printers and off-the-shelf components for the first time. This allows quick and inexpensive electrode manufacturing and opens the possibility of creating electrodes that are customized for each individual user. Our 3D printed electrodes are compared against standard wet electrodes, and the performance of the proposed electrodes is suitable for BCI applications, despite the presence of additional noise.

## 1. Introduction

**Figure 1:**
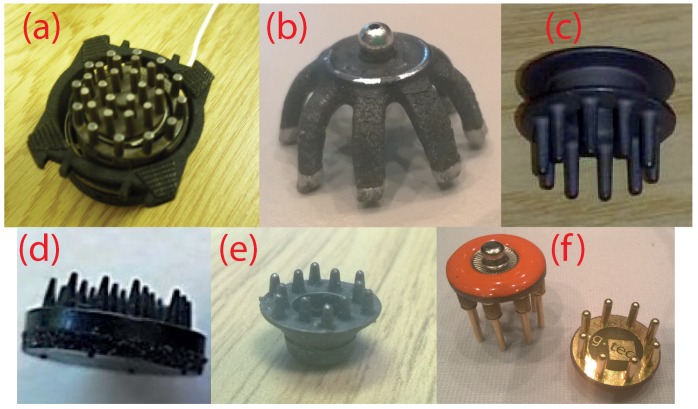
Examples of current dry fingered EEG electrodes. (**a**) Wearable sensing [4]; (**b**) Cognionics [5]; (**c**) Neuroelectrics [6]; (**d**) IMEC [7]; (**e**) Florida Research Instruments [8]; (**f**) g.tec g.SAHARA [9].

Electroencephalography (EEG) is the monitoring of a person’s brainwaves by placing small metal electrodes on the scalp and is of key use in epilepsy diagnosis, stroke rehabilitation and other healthcare applications [1]. Traditional EEG systems are significantly limited by the requirement to have a wet gel added to the electrodes, which is used to make a good contact between the electrode and the scalp through hair. This water-based gel, with an added ionic compound, such as sodium chloride, takes a long time to apply; leaves a mess; dries out over time; and is highly unpopular with patients, users, researchers and clinicians. In recent years, a significant number of approaches towards dry EEG electrodes have been proposed [2,3], and several are now available commercially (Figure 1). Generally, these are based on electrodes with fingers that can penetrate the hair and make direct contact with the scalp. As such, they are formulated so that no skin preparation or conductive gels are required, significantly improving the setup time and the user’s comfort compared to traditional wet EEG electrodes.

However, while there is much interest in dry electrode technology and many of the attempts to develop such electrodes show promising results, dry electrodes are still not in widespread practical use due to a number of factors. These range from poor contact noise, to difficulties in keeping the electrodes attached, to the substantial costs required to purchase new electrodes. Significant work on next generation electrodes is still required, and [2] notes that “the ultimate solution will likely be a combination of some circuit design, but even more a matter of innovative mechanical construction and signal processing” and that “efforts directed in that direction are expected to yield significant returns for this field”.

This paper presents our work on the use of 3D printing to rapidly manufacture dry EEG electrodes. As a new and readily available manufacturing approach, our method provides a way for individual researchers to make their own electrodes in an easy and accessible way, going beyond the electrode configurations that are currently available at some cost commercially. Many open source EEG hardware projects are currently under way, for example OpenBCI [10], and these have already investigated 3D printing for manufacturing and customizing the EEG headset and its connectors. Our work extends this and allows 3D printing of the EEG electrodes, as well. As such, the electrodes can be manufactured very inexpensively and on the spot, giving much easier access to dry EEG technology. We have made our design files available under an open source license to facilitate this.

Further, the complete manufacturing process for the 3D printed EEG electrodes takes less than 90 min and opens new possibilities in the personalization of the electrodes for each individual. Our electrodes are fingered for easy penetration through hair, and it is possible to change the tip diameter, tip profile and finger length to best penetrate through different types of hair on an individual-by-individual basis in near real time.

We have investigated two approaches for 3D printing rigid dry EEG electrodes: one using printed plastic mixed with a carbon-based conductive compound to give an intrinsically-conductive electrode that can be used immediately after printing; and one using standard printed plastic to which a conductive coating is applied to make it usable as an EEG electrode. The coating process increases the time required to make the electrodes (due to the coating drying time) and means we are still some way from closed-loop personalized on-the-spot manufacture. Nevertheless, to date, it has obtained significantly superior electrical and mechanical performance and avoids the bio-compatibility issues with carbon nano-particles present in conductive 3D printing filaments and, so, is our currently preferred method. In this paper, Section 2 overviews the materials chemistry that informs our manufacturing and electrode design; Section 3 presents our electrode designs and the manufacturing process; while Section 4 gives a detailed performance characterization using an EEG head phantom; finally our results are discussed in Section 5, which also overviews our progress towards printing electrodes using conductive plastic materials.

## 2. Electrode Materials and Chemistry

Key to the quality of the signal from an EEG electrode, whether wet or dry, is the electrochemical properties of the material and coating that are used to form the electrode. Understanding how these properties can improve or worsen the EEG measurement and how they interact with the connection to the skin is essential for the development of dry electrodes and analysing their performance. This section briefly overviews these properties for both wet and dry electrodes in order to inform the choice of materials available for printing in Section 3.

### 2.1. Wet Electrodes

**Figure 2:**
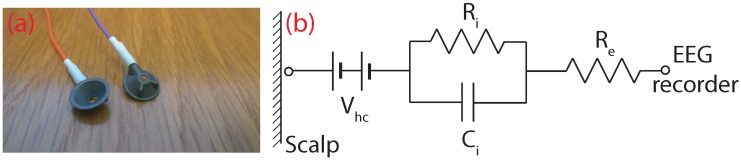
Current wet EEG electrodes. (**a**) Disposable Ag/AgCl electrodes are hollow cups to hold the conductive gel; (**b**) equivalent electrical circuit.

Wet EEG electrodes, such as the ones shown in Figure 2a, are filled with an electrolytic (conductive) gel and put into a cap or glued to various positions along the scalp in order to make an electrical connection with the human body. In essence, the electrode is a transducer that converts ionic currents coming from the human body into electron currents that can be measured by conventional electronics. The electrical equivalent circuit of a wet electrode is shown in Figure 2b. $V_{hc}$ represents the half-cell potential of the electrode, which occurs in the presence of charge carriers (ions) concentrated at the electrode-electrolyte interface and which causes a long-term drift in the recorded potentials [11]. $R_{i}$ and $C_{i}$ are parameters that determine the impedance of the electrode-electrolyte interface, and $R_{e}$ specifies the material resistance of the electrode [11].

Standard wet electrodes are made from a silver (Ag) base coated with silver chloride (AgCl), which is then sintered (heated below the meting point) to improve the electrical properties. These silver/sliver chloride (Ag/AgCl) electrodes offer excellent electro-physiology recording properties. Firstly, Ag/AgCl electrodes have a very low half-cell potential, meaning better drift and noise performance at DC and low frequency measurements [11]. They are also non-polarisable, which gives them long-term stability and lower susceptibility to movement artefacts [12]. Polarisation is an undesired effect, which occurs when there is a build-up of charge carriers at the electrode-electrolyte interface, which acts as an insulating barrier, such that current is no longer able to pass.

In addition to the electrode material, for good quality recordings, a conductive gel and a skin preparation stage are required. The gel serves as a conductive medium for the ionic currents coming from the body, as it has an abundance of Cl-ions. It also maximizes the skin contact area (even through hair), effectively lowering the contact impedance of the electrode, and acts as a mechanical buffer maintaining contact with the skin during movement of the electrode. To complement the gel, skin abrasion, removing parts of the epidermis, is often performed before EEG experiments. This lowers the electrode-scalp contact impedance, but it is unpleasant to users and poses a risk of infection [13]. High quality EEG recordings can be recorded using modern instrumentation even with contact impedances higher than 40 kΩ [13], although total impedances below 5 kΩ are still the target for clinical practice [14].

### 2.2. Dry Electrodes

Without the conductive gel present in a dry electrode, it is the material on the surface of the electrode that dominates the performance in terms of noise and contact impedance. Particularly challenging is the recording of low frequency components, which are contaminated by both low frequency 1/*f* noise and drift in the half-cell potential. The work in [15] characterized seven different electrode materials (tin, silver, sintered Ag/AgCl, disposable Ag/AgCl, gold, platinum and stainless steel), and the different performances are summarized in Table 1. Ag/AgCl has the best performance for low noise, low frequency measurements, but silver and gold-plated silver electrodes also provided results good enough for practical EEG recordings. For example, the low frequency drift of silver electrodes stabilized after approximately 15 min, somewhat longer than the one minute for Ag/AgCl, but still feasible, especially given the large input ranges of modern amplifiers, which allows recording despite large drift values. Another study on electrode coatings [16] confirmed that the low frequency drift rate decreases over time, such that silver electrodes eventually come to a stable potential.

As a result, dry EEG electrodes have been reported using a number of different materials for the body contact. To our knowledge, the first occurrence in the literature of dry EEG electrodes is a paper from 1994 [17] (although references to dry ECG electrodes for heart monitoring date back to the 1960s [18]). The work in [17] presented a dry active electrode for EEG recordings based on a 3-mm steel disk coated with nitride on one side and an impedance converting amplifier on the other. This new electrode was compared with wet Ag/AgCl electrodes for different evoked potentials, and the authors state that it had a similar performance. The paper briefly discusses the low frequency motion artefacts present with the dry electrodes and suggests that they can be removed by reducing the size of the electrode.

Since then, a wide number of materials have been considered for making dry EEG electrodes [19,20,21,22]. The commercially available g.SAHARA electrodes [9,23] use gold and a similar closely-positioned impedance-converting amplifier approach. The work in [23] clearly shows that such electrodes are suitable for brain-computer interface applications with some disadvantages in the lower frequency (<3 Hz) performance. The work in [24] used a conductive polymer rather than metal and showed that the electrode impedance varied linearly with the surface contact area and that the polymer electrodes had better long-term stability in comparison to wet electrodes.

Although the fingered electrode approach (Figure 1) is the most common, a number of alternative electrode shapes has been considered and combined with novel materials to form dry electrodes. The work in [25] used multiple small Ag/AgCl bristles rather than fingers to connect to the skin. Most test subjects reported that the bristle type of electrode was more comfortable than rigid pin electrodes, and compared to gel-based electrodes, the bristle electrodes were found to have similar performance in the 7–44-Hz frequency range. The work in [26,27] presented microneedle-based electrodes, which penetrate the outer layer of the skin in order to make a better contact. The work in [26] found that silver chloride (AgCl)-coated needles achieved better contact impedance than silver-only needles of the same type, although they were designed for usage on the forehead, which has no hair, and this makes them impractical for other hairy locations along the human scalp essential for EEG. The work in [27] used an array of carbon nanotubes as the skin-penetrating needles. The performance of these dry electrodes was similar to wet electrodes, but again, they were placed at non-hairy sites, and no comments were made regarding the usability of microneedle-based electrodes for haired sites. In addition, the authors state that the bio-compatibility properties of carbon nanotubes are unknown and that further research is needed regarding the safety aspects of carbon nanotubes.

To our knowledge, 3D printing of EEG electrodes has not been considered in detail before. One online publication [28] indicates that a printed conductive polymer can be used to acquire EEG signals using a fingered electrode approach with gel, but no details on the design, implementation, manufacturing or performance are given. The work in [29] used a high resolution 3D printer to make a microneedle-type electrode. This dry electrode contained 180 needles with 250-μm spacing, which were 3D printed and then coated with gold to achieve conductivity. Again, they were tested only at the non-hairy Fp1 and Fp2 EEG sites.

Our work presents the design and performance of fingered dry 3D printed EEG electrodes for the first time. We specifically restrict the manufacturing to desktop-grade 3D printers in order to give a low cost and readily accessible process for users to make and customize their own electrodes.

## 3. 3D Printed Electrode Design

### 3.1. Manufacturing Overview

We investigated 3D printing of EEG electrodes because it offers substantially quicker and more accessible manufacturing than traditional moulding or milling techniques. As a rapid manufacturing technique, it gives flexibility, so that many different design iterations can be developed, tested and improved. For the purpose of EEG electrodes, this also means that different electrodes with various parameters, such as the number of fingers and tip sizes/shapes, can be tailored to each different person on demand. In particular, we have considered only desktop-grade 3D printers in order to allow inexpensive and easy access to the electrode manufacturing process. A consumer-grade Ultimaker 2 printer was used with a 20-μm layer resolution and position precision on the *X*/*Y*/*Z* axes of 12.5/12.5/5 μm. A downside of this 3D printer is that it uses a Bowden-type feeder, which was problematic when attempting to print particularly brittle materials (see Section 5).

Our 3D printing was done in Fab Lab Manchester (Manchester, UK) [30], which is a digital fabrication space that is free for use by the public, and all of our reported electrodes were printed at no cost. When submitted to 3D HUBS [31], a worldwide site that can be used to solicit tenders to make a printable design in a nearby location, quotes for manufacturing were approximately £13.50 for 100 electrodes.

### 3.2. Mechanical Design and Printing

**Figure 3:**
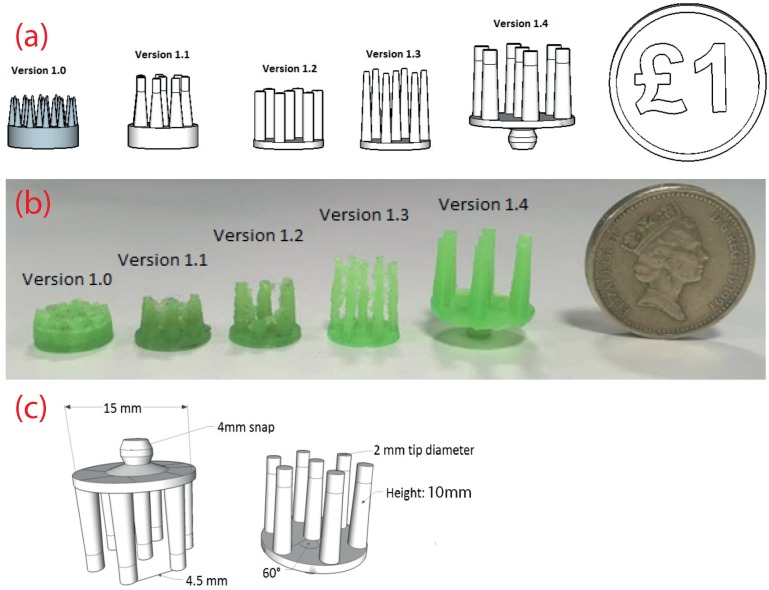
Design of the 3D printed electrodes. (**a**) Evolution of the design for creating robust to manufacture shapes; (**b**) evolution of the printed output as the printer settings were optimized; (**c**) detail on the final electrode design.

Our fabrication process first prints the mechanical structure of an electrode using standard, non-conductive, 3D printer plastics, and second, we coat these by hand with a suitable conductive material to make the final EEG electrode. The design starting point is thus a mechanical model of a fingered electrode, which has long and thin prongs, which penetrate the hair and achieve contact with the skin, similar to the electrodes in Figure 1. Multiple versions of a basic fingered design were considered (Figure 3a) in order find suitable settings that produced high quality mechanical devices after printing. Five different design iterations are presented in Figure 3a together with a UK £1 coin for comparison. The electrode diameter of Versions 1.0–1.3 is 10 mm, and the length of the prongs is 4 mm, 7 mm, 7 mm and 10 mm, respectively.

Version 1.4 represents the final base version of our electrodes and is shown in Figure 3c in detail. This is used as the starting point, which can be customized in terms of the prong number, diameter and similar to form different electrode configurations (see Section 4). For Version 1.4, the base diameter was changed from 10 mm to 15 mm in order to allow a future active circuit to be fitted on top. It also features a printed 4-mm snap connector so that it can be easily connected to standard EEG or ECG equipment. The tips of the prongs have a diameter of 2 mm, and the total height is 10 mm, so that contact remains good even through thick hair.

In parallel to the mechanical design process, each iteration was 3D printed using standard PLA (Polylactic Acid) plastic. The first four versions (1.0–1.3) were trial versions to find suitable dimensions for electrodes and also to understand the 3D printing capabilities of the Ultimaker 2. The best 3D prints of each version are shown in Figure 3b. Version 1.0 clearly shows that the 3D printer cannot print the narrowly-spaced fingers with the default ultra-high quality settings. Version 1.1 and Version 1.2 present an improvement in the print quality due to the modified 3D model; however, the quality of the print is still not satisfactory. It can be observed that there are strings between each prong, which is an undesired effect called stringing and occurs when the printer head moves from one finger to another and creates small bridges between the fingers due to the presence of leftover material in the extruder tip. Version 1.3 has a slight improvement in the print quality with the stringing effect reduced by using a retraction technique. Before the printer head moves from one position to another, the leftover material present in the tip of the extruder is retracted (drawn back in), and then, the printer head is moved to the new position. Version 1.4 is again the final version and has the best printing quality. The stringing effect is eliminated by using the retraction technique and increasing the printer travel speed, meaning that the strings do not have time to solidify. It can be seen that the surface finish is very fine with the exception of the electrode base, where a base support was present during printing. (One downside of extrusion-based printers, such as the Ultimaker 2, is their inability to print horizontal objects. This meant that an additional mechanical support structure had to printed below the base of the electrodes and removed after the printing process.) The total printing time varied depending on the settings used and was approximately 20 min.

Once the design had been finalized, the parts were also printed using an ABS (Acrylonitrile Butadiene Styrene) plastic. The PLA plastic is slightly more flexible, which gives better stability of the electrode fingers, whereas some of the fingers of the ABS printed electrodes snapped off occasionally. These current electrodes are thus rigid. This is a common approach to dry electrodes, with the g.tec and Enobio dry electrodes in Figure 1 using similar structures. Cushioning could/should be provided by the headstage used to hold the electrodes in place, and for example, the OpenBCI headstage has springs connected to the electrodes. This said, rigid prongs could present a potential safety issue if an abrupt force were applied, and for prudence, we would recommend that the electrodes be only used while sitting, for example in standard brain-computer interface paradigms. In addition, due to the rigidness of the electrodes, if the pressing angle is not right toward the electrode, some of the prongs make better contact than others. A detailed study on the noise contribution of each individual prong contact is described in [32] and, thus, was not investigated here.

The design files for each of our five printed configurations (see Section 4) are available on GitHub (San Francisco, CA, USA) under a Creative Commons Attribution-ShareAlike 4.0 International license. The files can be cloned using the Git or SVN tools and the URL: https://github.com/CASSON-lab/3d_printed_eeg_electrodes.git. Use of the designs should be accompanied by a citation to this publication and a link to the GitHub page.

### 3.3. Conductive Coatings

State-of-the-art EEG electrodes have either a silver base with AgCl coating or have a sintered Ag/AgCl coating, as discussed in Section 2. Applying a sintered Ag/AgCl coating requires access to a specialist laboratory and chemical processes and, so, is not suitable for our aim of low cost easy to manufacture electrodes. It is possible to purchase Ag/AgCl ink (see Table 2) and use it similar to our approach below, but this is expensive and again not in-line with our current aim. Instead, to make the electrodes conductive and suitable for electro-physiological recordings, we coated them with silver. This gives a reduction in performance compared to Ag/AgCl, but with a much easier manufacturing process.

There are several solutions for silver conductive coatings on the market, summarized in Table 2. Most options are either expensive or difficult to apply with the exception of pure silver paint, which costs £10 for 3 g and can be applied by hand simply using a paint brush. Before the silver paint was applied to the electrodes, the safety datasheet [37] was carefully examined in order to determine any potential hazards when applying the paint and also to determine its bio-compatibility. The following materials were listed:

- Silver: Metallic element often used in biomedical applications, which has no safety implications to humans [39].
- 1-Ethoxypropan-2-ol: Commonly-used organic solvent labelled R10 and R67 under the European Union Regulation No. 1272/2008 for hazardous materials [40]. R10 means that the element is flammable, and R67 means that the vapours may cause drowsiness and dizziness.
- Ethanol: Often used as an antiseptic or solvent. Labelled R11 meaning that it is highly flammable.
- Acetone: Organic solvent often used in the cosmetics industry. Listed as R11 (highly flammable), R36 (can cause eye irritation) and R67 (vapours may cause drowsiness and dizziness). Repeated exposure may cause skin dryness or cracking (R66).
- Ethyl acetate: Solvent that has the same hazardous labels as acetone.

No other health hazards were listed in the safety datasheet, and it also states that “this substance has no evidence of carcinogenic properties” [37]. It is important to note the R66 label regarding skin irritation, which is relevant to the solvents present in the paint. Both acetone and ethyl acetate are used as nail polish remover. They evaporate quickly, even at room temperature, meaning that no hazardous substances will be left on the coating when it is dry. The silver paint is thus bio-compatible, but the electrodes must be left for a sufficient drying time after coating, approximately one hour, before attempting to use them.

**Figure 4:**
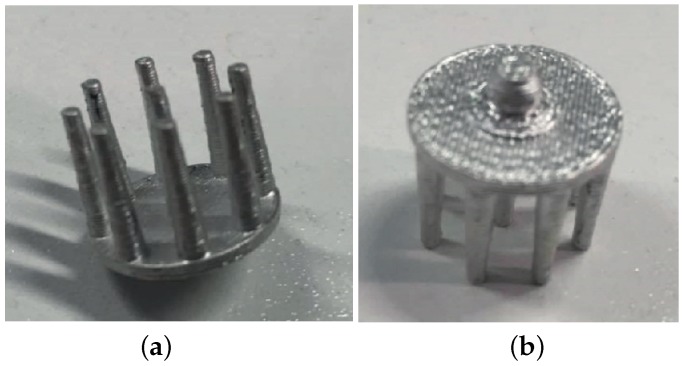
Example of a 3D printed EEG electrode coated with silver paint. (**a**) Underside showing fingers for penetrating the hair; (**b**) Top side showing 4 mm snap connector.

To perform the coating, the silver paint is applied using a very fine paint brush and left to dry for one hour. Approximately, 1.5 g of silver paint are sufficient for the coating of 10 electrodes, and just one layer of coating provides good conductivity levels. The surface area of one of our electrodes is on average 957 mm^2^, giving an approximate coating thickness of 30 μm. An example of a final printed and coated electrode is shown in Figure 4. To confirm the coating, the DC resistance between the prongs and the snap connector was measured to be approximately 0.4 Ω, which compares favourably with measurements of a commercial dry EEG electrode (approximately 0.3 Ω) and for a standard Ag/AgCl electrode (approximately 0.4 Ω).

**Figure 5:**
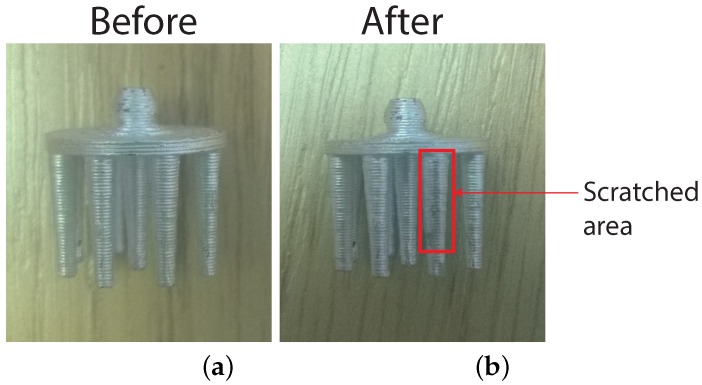
Electrode coating before and after 100 finger scratches. (**a**) Sample before test; (**b**) Sample after test shows some small areas of coating removed.

In our experience from everyday use, none of the electrodes have yet become non-functional due to the coating coming off (through finger scratching, wear and tear or otherwise). In order to provide a quantitative assessment, we have performed a finger scratch test on one electrode prong, which represents a balance between mild scratching on the head and scratching with a sharp object. No visible marks were found after 50 finger scratches; after 100 finger scratches, there were several tiny spots without coating, as can be seen in Figure 5b. The scratch test was performed on 10 different electrode prongs, and similar results were observed after approximately 100 scratches. If coating removal were to occur after actual usage, the base material is a different colour, so that missing coating is easy to spot, and new silver can equally be easily painted on. If necessary, a second layer of coating or curing at approximately 150 °C for 10–15 min will provide better adhesion to the plastic without melting it. In addition to the scratch test, the electrodes were sterilised in a solution of Dettol for 5 min, which is a common procedure in academic EEG labs. The silver coating remained intact, but further clinical autoclave-type cleaning has to be investigated.

## 4. Performance Characterization

### 4.1. Overview

To systematically characterize the electrode properties, our printed and coated electrodes were connected to an EEG head phantom made using gelatin with conductive saline solution, similar to that reported in [41,42,43]. This allows controlled and repeatable testing with a known input signal. Many dry electrode development papers only consider functional testing, placing wet and dry electrodes on a subject’s head and showing that they record similar signals. However, this does not capture the electrode properties (noise, impedance and similar) and can be highly inaccurate. The work in [44] showed that correlation coefficients over 0.9 can be achieved even if there is more than 15 μVrms of differences due to noise present between the two electrodes. The use of a head phantom and measurement of the electrode properties is therefore the preferred testing methodology.

In order to understand how the different electrode parameters affect the performance, five different configurations of 3D printed electrodes (rigid, Version 1.4) with variations in the tip number/size were made. Only one electrode sample pair of each variation was tested, as our goal was to demonstrate that EEG signals can be recorded successfully and not to explore the statistical variations between samples of the same configuration due to the manufacturing process. The different configurations of our 3D printed electrodes are summarized in Table 3. These are compared to a commercially-available Ag/AgCl dry electrode from Neuroelectrics (Drytrode) [6] and to a conventional Ag/AgCl wet electrode connected with EC2 conductive gel [45]. In all cases, two electrodes of the same configuration were connected to the head phantom and used to measure the contact impedance, contact noise and measurement of played-in synthetic EEG signals. The signals were recorded at 250 Hz using a custom system based on the TI ADS1299 EEG amplifier.

### 4.2. Impedance

**Figure 6:**
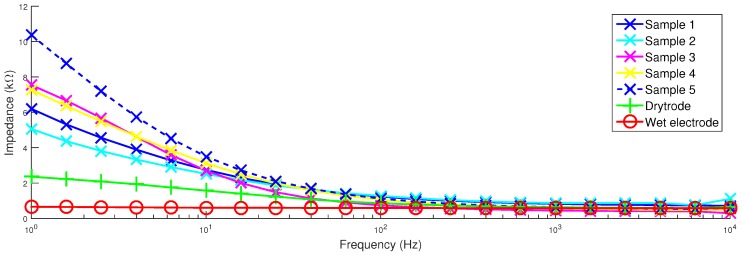
Contact impedances of the different electrode samples.

Figure 6 shows how the contact impedance of the electrodes varies with frequency. It can be seen that the wet electrodes provide the best performance with impedances below 1 kΩ in all cases. This is followed by the Drytrode from Neuroelectrics, which is below 2 kΩ for all but the lowest frequencies. At low frequencies, an increase in the impedance is seen and expected due to the lack of polarization effects in the Ag/AgCl electrode coating, as suggested in [11]. Our electrodes have higher impedances, approximately 2.5 kΩ; below the 5 kΩ target. The impedances of all of the electrodes converge at higher frequencies.

**Figure 7:**
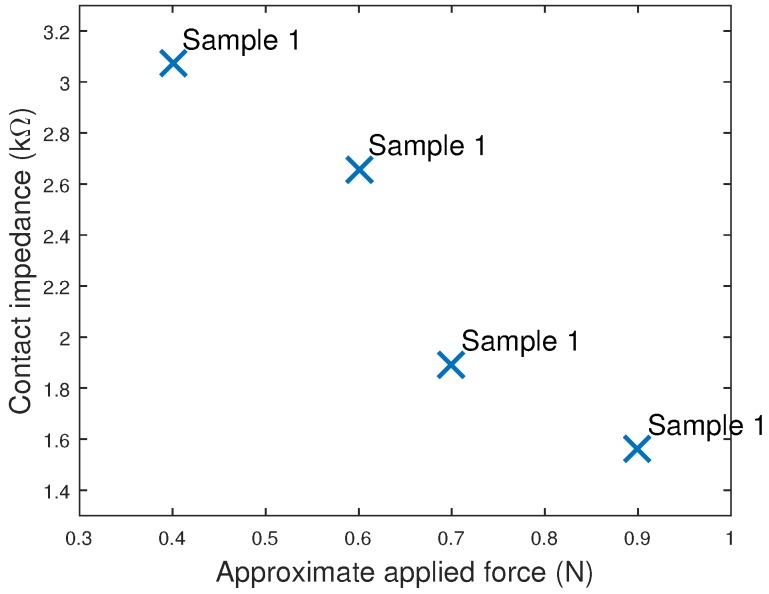
Contact impedance of Sample 1 when held against the head phantom with different levels of force.

When using our head phantom, the contact impedance was found to vary with the force applied between the electrode and the phantom. The effect was found to be largest for Sample 1, which had the lowest number of fingers, and the impedance at 15 Hz for Sample 1 at difference applied forces is shown in Figure 7. Lower impedances could be obtained if the electrodes were pushed down more forcefully, although this will come at the cost of user comfort. EEG headsets, such as OpenBCI [10], are beginning to include springs to regulate the force of the electrodes on the scalp, which will minimize this effect and can potentially be used to help control and achieve acceptable impedances.

**Figure 8:**
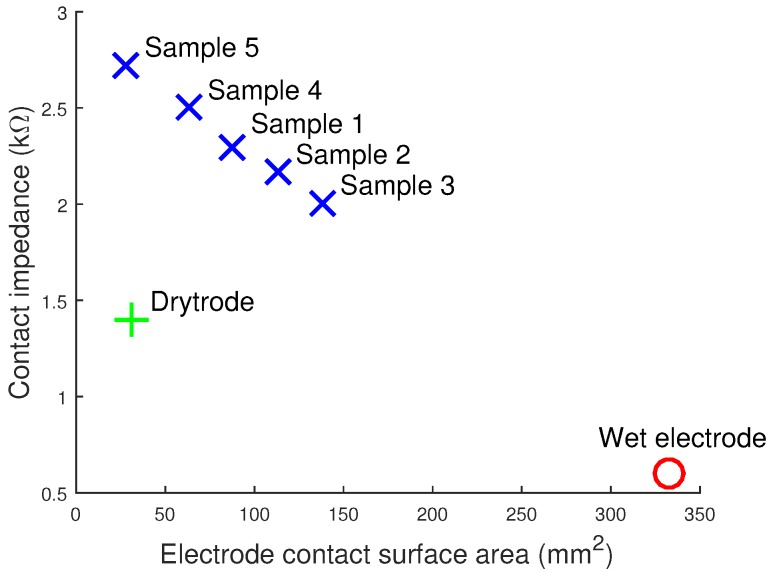
Contact impedance of printed electrodes decreases as the contact area of the 3D printed electrodes increases.

In addition, the electrode impedance was found to decrease with the surface area of the electrode. Figure 8 plots the contact impedances of our five samples and shows that there is an approximately linear relationship with electrode area. While our 3D printing allows the easy customization and changing of tip sizes and areas, care must be taken when doing this to avoid overly increasing the electrode impedance.

### 4.3. Contact Noise and Drift Rate

Low level noise in EEG recordings is present due to a number of sources, including the electronics of the EEG amplifier and due to the electrode connection itself [16]. Contact noise when no signal is present provides insights into the electrode performance at low frequencies, the drift rate and susceptibility to power line interference. To measure these factors, for each electrode configuration, two electrodes were connected to the head phantom, but no signal played in. Instead, the residual noise present was recorded, which will come from both the electrode and the amplifier, with the amplifier noise properties being common across all tested electrodes. For each electrode configuration, a 2 min recording of the contact noise was taken and analysed in three ways:

- The root-mean-squared (RMS) contact noise was calculated by splitting the recording into ten-second segments, the RMS of the signal present between 0.1 Hz and 40 Hz found for each segment and, then, the average and standard deviation found across segments.
- The drift rate was calculated by splitting the recordings into 60-s segments, then a Butterworth low pass filter was used to extract low frequency (<0.16 Hz) signal changes, and the results were averaged to give the drift rate.
- The powerline noise was estimated by taking the FFT of each 10-s segment and finding the average power of the signal present at 50 Hz.

For all three of the measures, the average results and standard deviations are shown in Table 4.

**Figure 9:**
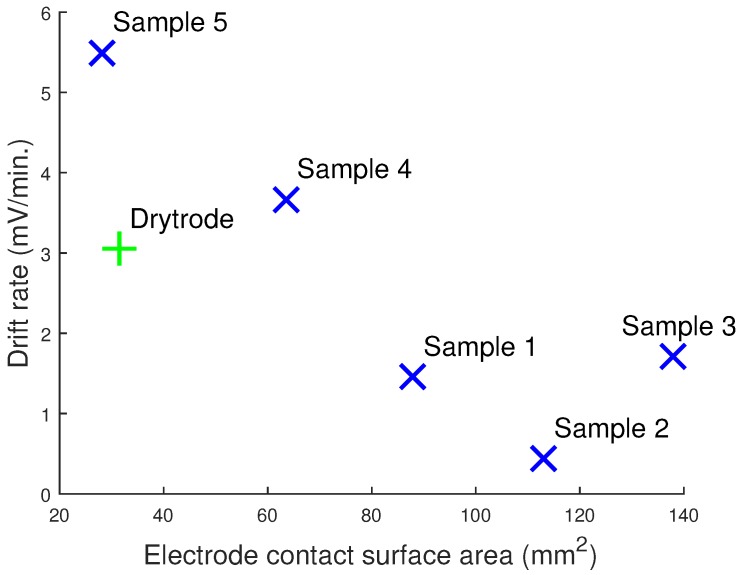
Drift rate of the printed electrodes decreases as the contact area of the 3D printed electrodes increases.

All of the dry electrodes have a large drift rate in comparison to the wet electrodes. Further, as with the contact impedance, Figure 9 shows that the drift rate of the dry electrodes decreases with the electrode contact surface area. In the literature, drift rates are usually related to the metallic properties of the electrodes. Our results clearly show that there is also a relationship between the contact area and the low frequency drift. Electrode impedance is also proportional to the contact area for the 3D printed silver electrodes; however, this is not true for the dry Ag/AgCl electrodes, which have lower contact impedances despite the lower contact area. This suggests that the drift rate may be uncorrelated to the electrode impedance and instead dependent on the contact area and metallic properties. Drift rates are usually not a problem for modern day instrumentation, which have wide input ranges and because the drift rate usually declines after the electrode potentials have stabilized [16]. However, not many studies have explored the drift rates of dry electrodes, and a constant drift rate could lead to a saturation of the input amplifier or ADC eventually if they are d.c. coupled.

Samples 3 and 4 have high levels of contact noise, over 15 μVrms, and so are unlikely to be suitable for use in practice. In contrast, the other three samples have noise levels of approximately 5 μVrms and compare reasonably with the previously-reported noise values for silver-coated electrodes: 3.3 μVrms over 0.5–500 Hz [16]. As would be expected, the noise spectrum for our electrodes shows that the majority of the RMS noise is present at lower frequencies (<10 Hz) and follows a 1/f trend. The thermal noise contribution to the total RMS noise due to the contact impedance was found to be negligible (0.08 μVrms for 2.5 kΩ impedance). Further, no correlation was found between the RMS noise and contact impedance. The RMS noise was also independent of the contact surface area of the electrodes.

Unless it leads to saturation of the EEG amplifier, powerline noise is not usually a problem for EEG recordings, as in most applications, it lies outside the frequency range of interest, and it can be easily removed after recording using a digital notch filter. Nevertheless, for our electrodes, the powerline contribution to the noise is low, approximately 1 μVrms. This is again in-line with values previously reported in the literature for wet electrodes [13].

### 4.4. Synthetic Signal Testing

To demonstrate the collection of EEG signals in a controllable manner, five different sinusoidal signals were generated and fed into the head phantom in order to produce EEG signals on the phantom surface. EEG recordings were 60 s in length and were split into six different parts, 10 s each. These were filtered using a 0.1–45 Hz bandpass filter and the MATLAB normalized correlation function xcorr used to calculate the correlation of the dry electrode recordings with wet electrode recordings. The average and standard deviation of the dry vs. wet signal correlations at five different input frequencies are presented in Table 5 for our six dry electrodes (five 3D printed, one commercial). This shows systematically how well the printed electrodes detect EEG components at different frequencies. A correlation of one means that the two signals are completely identical.

Table 5 shows that in many cases, there is a good correlation (>98%) between the 3D printed dry electrodes and conventional wet electrodes. The two best performing pairs are Samples 2 and 5 due to the reduced amount of RMS noise present in these two designs, as previously shown in Table 4. Typical correlation values reported for previous dry fingered EEG electrodes are: >0.93 [46]; 0.89 [47]; 0.83 [48]; 0.81–0.98 [49]; 0.68–0.90 [50]; 0.39–0.85 [51]; and our values compare very favourably with these.

### 4.5. Functional Testing

Finally, to demonstrate the collection of actual EEG signals, Sample 1 was connected to one volunteer and used to measure alpha waves. EEG alpha waves correspond to activity in the visual cortex of the brain and are found in the frequency range 8–12 Hz. EEG alpha waves can be modulated by opening and closing the eyes (more alpha is present when the eyes are closed), and so, they give an easily identifiable signal structure to demonstrate that true EEG is being detected by the 3D printed electrodes.

Two 3D printed dry electrodes were attached to the O1 (hairy) and A1 (non-hairy) positions according to the 10–20 system using an adjustable elastic band and surgical tape, respectively. The subject was asked to stay with their eyes open and then to close them in order to induce alpha activity. To provide a comparison case, a conventional wet electrode with EC2 gel was connected at the same time in the O2 position. This shared the same A1 printed electrode reference. Being from a different position on the head, the collected EEG signals are not expected to be identical, but both should collect the same information on the induced alpha activity. The signals were recorded at 1024 Hz using a CamNtech Actiwave biomedical waveform recorder.

**Figure 10:**
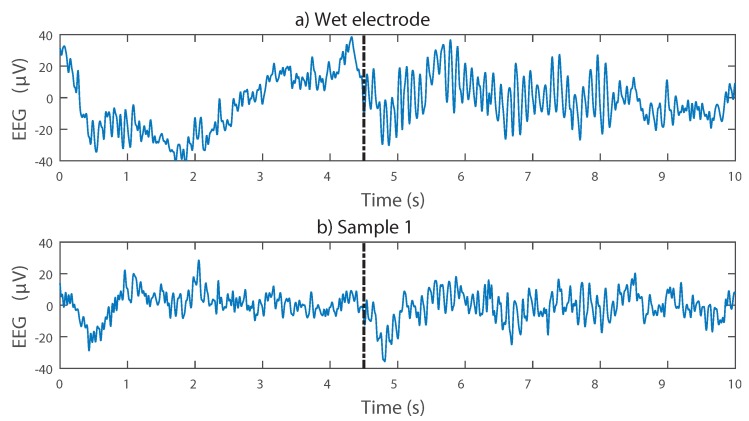

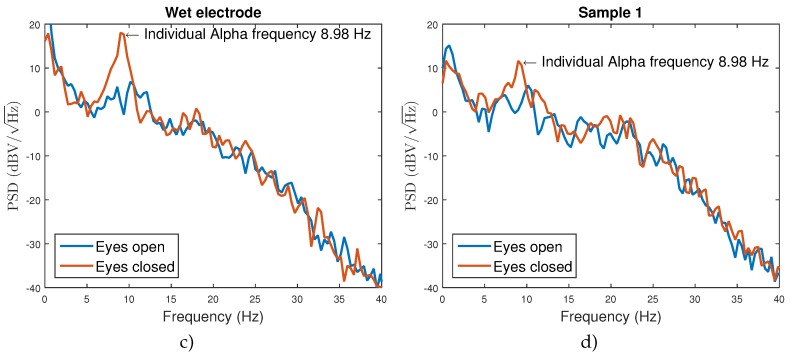
Alpha activity recorded using wet and 3D printed EEG electrodes. Note that the wet electrode was placed at O2 and the dry electrode at O1, and so, the two time domain traces are expected to be similar, but not identical. (**a,b**) Time domain signals. The emerging of alpha activity due to eyes being closed is approximately marked with the black dotted line; (**c,d**) frequency domain signals. The extracted individual alpha frequency is the same using both electrodes.

Figure 10 shows example recordings, demonstrating that the presence and absence of alpha activity depending on the subject’s eye state can be detected with the dry 3D printed electrodes. The Welch’s power spectral density estimate was calculated in MATLAB using a 2-s window size and 65,536 DFT (Discrete Fourier Transform) points. The peak in the frequency domain due to alpha activity is easily seen, and with both the wet electrode and dry electrodes, the individual alpha frequency (the frequency of the maximum alpha activity) is extracted as the same frequency. A reduced signal-to-noise ratio is presented, with the alpha peak being only about 5 dB above the background activity when using the dry electrode, compared to approximately 15 dB when using the wet electrode. This is to be expected given the noise results from Table 4 and does not prohibit the alpha peak from being easily discernible. Although our electrodes are printed, silver based and have no conductive gel, they are detecting the same brain-related information as the wet electrode.

## 5. Discussion

We have demonstrated rigid EEG electrode prototypes fabricated using a desktop 3D printer and coated with off-the-shelf silver ink for quick and low cost manufacture. These prototypes were functionally tested for EEG signals and show that alpha rhythms can be correctly recorded. The electrode properties, and particularly the noise and impedance, have also been characterized. While these properties are generally worse than using traditional wet Ag/AgCl electrodes, our 3D printed electrodes still produce a usable signal and offer the potential for rapid manufacturing and customization to each user. We anticipate doing this by varying the electrode tip diameters, profile (flat/sloping/rounded) and the length of each finger to best penetrate through each person’s hair. The impedance (Figure 8) results show that care must be taken when doing these customizations, as different tips will directly affect the electrode properties, and the next step in our work will be to build a library of electrode shapes that balance different customizations with electrode performance, as an intermediate step before fully custom-making electrodes for each individual person.

If superior performance is required, while we have focused on the fabrication of dry electrodes in order to give a quick and easy setup, our manufacturing does not preclude the use of a gel with the electrodes. If desired, a hole could be added to the top of the electrode, and this used to add gel during the electrode setup, turning our dry 3D printed electrodes into wet 3D printed electrodes. Further, our present approach 3D prints the mechanical structure of an electrode that is then coated with a suitable material for optimizing the EEG sensing. Rather than using our silver coating process, for superior performance, an Ag/AgCl coating process could be used, using the Ag/AgCl ink noted in Table 2.

Regardless of the ink used, the coating process will always add to the time required for manufacturing, and this means that we are still some way from closed-loop personalized on-the-spot manufacture. The next stage for this research is to 3D print directly using a conductive material such that the electrodes could be used immediately after printing, without requiring a coating step. Suitable materials are relatively new to the market, and at the time of the research, only five different conductive options were available (summarized in Table 6). These conductive filaments are based on ABS or PLA plastic mixed with a carbon-based conductive compound. As there is a high concentration of plastic in the mixture, the volume resistivity of the materials is high and is not comparable to metals or metal-coated objects (silver has a resistivity of 1.59 × $10^{-10}$ Ωcm).

To date, we have tried several of these materials, but they are extremely brittle and very often break during the printing process between the Bowden feeder and the extruder. High quality 3D electrode prints, suitable for further investigation, have not been yet achievable. The main reason behind that is that a 3D printer with a Bowden drive instead of a direct drive was used. This was a feature of our desktop-grade printer, although alternative models without a Bowden drive, such as the Makerbot Replicator [57], are available. We highlight it here as an important area to research in the future, and particularly, if the manufacturing is not restricted to using a low cost or a Bowden-type 3D printer, a completely closed-loop manufacturing of EEG electrodes could potentially be achieved. However, it is important to note that the bio-compatibility of conductive printable materials is unknown, and it should be further investigated before performing trials on humans.

## 6. Conclusions

This paper has presented a novel methodology for the fabrication of 3D printed EEG electrodes. These allow a user to go from a CAD design to a functional prototype in less than 90 min only using a desktop 3D printer and off-the-shelf components. It therefore holds substantial potential for personalized healthcare, with custom electrodes made on the spot for each individual. Performance results show that the fabricated electrodes can measure EEG signals, and the performance characteristics have been fully reported. The 3D printed electrodes were compared with conventional wet electrodes, the bench tests showed that the contact impedance and noise floor for the dry electrodes were higher, which is in-line with other similar studies. The functional test shows that despite the worse electrical characteristics, the 3D printed electrodes, at their current state, can be used for BCI applications, which do not require a high SNR. The presented methodologies simplify the fabrication and testing of electrodes, meaning that more time and effort can be spent on developing innovative electrode designs. To facilitate this, our hardware designs are available under an open source license from https://github.com/CASSON-lab/3d_printed_eeg_electrodes.git.

## Figures and Tables

**Table 1:** Summary of electrode performance using different materials for coating, based on [15].

Material	Offset Voltage, Resistance and Polarization	Rate of Drift	Noise Level
Sintered Ag/AgCl	Very low	Very low	Low
Disposable Ag/AgCl	Low	Very low	Low
Silver	Variable	Variable	Low
Gold-Plated Silver	Variable	Variable	Low
Platinum	Very high	–	Low
Stainless Steel	Very high	–	Medium
Tin	High	High	High

**Table 2:** Comparison of different silver coatings available.

Type	Resistivity (Ω/Square)	Quantity	Price	Comments
Silver tape [33]	0.3	–	£165	Adds much thickness
Silver epoxy [34]	<0.005	7 g	£70	Special curing needed
Silver pen [35]	0.02	8.5 g	£33	Difficult to apply
Flexible silver pen [36]	0.05	8.5 g	£52	Difficult to apply
Silver paint [37]	0.01	3 g	£10	Easy to apply
Flexible Ag/AgCl ink [38]	0.1	100 g	£280	Shipping £52

**Table 3:** Configurations of our 3D printed electrodes, the commercially-available dry Ag/AgCl (Drytrode) [6] and conventional wet electrode tested.

Sample ID	Number of Tips	Tip Diameter (mm)	Electrode Contact Surface (mm^2^)
Sample 1	07	2.0	87.9
Sample 2	09	2.0	113
Sample 3	11	2.0	138
Sample 4	09	1.5	63.6
Sample 5	09	1.0	28.2
Drytrode	10	∼1	∼31
Wet electrode	00	–	∼333

**Table 4:** Noise performance of different electrodes. Mean ± standard deviation.

Sample ID	Drift Rate (μV/min)	RMS Noise 0.1–40 Hz (μV)	Powerline Noise (μV)
Sample 1	1454	07.7 ± 1.1	1.2 ± 0.1
Sample 2	0438	04.9 ± 2.5	3.6 ± 0.3
Sample 3	1711	15.8 ± 3.5	1.0 ± 0.1
Sample 4	3665	16.2 ± 1.4	0.4 ± 0.0
Sample 5	5494	05.8 ± 1.3	0.8 ± 0.1
Drytrode	3046	00.5 ± 0.1	0.5 ± 0.1
Wet electrode	0126	00.4 ± 0.1	1.1 ± 0.1

**Table 5:** Correlation coefficients between dry and wet electrodes when measuring sinusoidal signals at different frequencies. Mean ± standard deviation.

Sample ID	Input frequency (Hz)				
	1	10	15	20	40
Sample 1	0.968 ± 0.003	0.955 ± 0.035	0.981 ± 0.003	0.982 ± 0.004	0.982 ± 0.003
Sample 2	0.954 ± 0.002	0.982 ± 0.004	0.988 ± 0.001	0.980 ± 0.010	0.988 ± 0.001
Sample 3	0.931 ± 0.011	0.862 ± 0.027	0.903 ± 0.011	0.923 ± 0.030	0.899 ± 0.086
Sample 4	0.924 ± 0.039	0.898 ± 0.032	0.926 ± 0.010	0.933 ± 0.019	0.931 ± 0.016
Sample 5	0.983 ± 0.002	0.981 ± 0.007	0.983 ± 0.003	0.989 ± 0.002	0.992 ± 0.001
Drytrode	0.989 ± 0.001	0.997 ± 0.000	0.992 ± 0.001	0.996 ± 0.000	0.995 ± 0.000

**Table 6:** Comparison of conductive 3D printing filaments currently available and suitable for use.

Supplier	Material	Conductive Compound	Volume Resistivity (Ωcm)	Price
Proto Pasta [52]	Conductive PLA	Conductive carbon black	15	£31
MakerGeeks [53]	Conductive ABS	Carbon fibre	10,000	£13
Functionalize [54]	Conductive PLA	Carbon nanotubes	0.75	£25
Graphene 3D Lab [55]	Conductive PLA	Graphene	0.6	£35
BuMat [56]	Conductive ABS	No information	1000	£31
